# Non-uniform salinity in the root zone alleviates salt damage by increasing sodium, water and nutrient transport genes expression in cotton

**DOI:** 10.1038/s41598-017-03302-x

**Published:** 2017-06-06

**Authors:** Xiangqiang Kong, Zhen Luo, Hezhong Dong, Weijiang Li, Yizhen Chen

**Affiliations:** Cotton Research Center, Shandong Key Lab for Cotton Culture and Physiology, Shandong Academy of Agricultural Sciences, Jinan, 250100 PR China

## Abstract

Non-uniform salinity alleviates salt damage through sets of physiological adjustments in Na^+^ transport in leaf and water and nutrient uptake in the non-saline root side. However, little is known of how non-uniform salinity induces these adjustments. In this study, RNA sequencing (RNA-Seq) analysis shown that the expression of sodium transport and photosynthesis related genes in the non-uniform treatment were higher than that in the uniform treatment, which may be the reason for the increased photosynthetic (Pn) rate and decreased Na^+^ content in leaves of the non-uniform salinity treatment. Most of the water and nutrient transport related genes were up-regulated in the non-saline root side but down-regulated in roots of the high-saline side, which might be the key reason for the increased water and nutrient uptake in the non-saline root side. Furthermore, the expression pattern of most differentially expressed transcription factor and hormone related genes in the non-saline root side was similar to that in the high-saline side. The alleviated salt damage by non-uniform salinity was probably attributed to the increased expression of salt tolerance related genes in the leaf and that of water and nutrient uptake genes in the non-saline root side.

## Introduction

It was estimated that 80 million hectares of the cultivated lands in the world were affected by soil salinity^1^. Excessive soil salinity can cause ion toxicity, osmotic stress, water and nutrient deficiency and therefore rapid reduction in growth of crops due to decreased photosynthesis^2–5^. Maintaining ionic homeostasis, balancing root water uptake and leaf transpiration and increasing nutrient uptake are critical for plants to cope with saline environments^4^. The extrusion of Na^+^ to the apoplast or external environment by salt overly sensitive (SOS) pathway proteins (SOS1, SOS2, and SOS3) or sequestration in vacuoles by vacuolar Na^+^/H^+^ antiporters (NHX) are two efficient ways to protect cells from Na^+^ injury^4, 6, 7^.

Salinity induced water deficit is caused by the imbalance between root water uptake and leaf transpiration^8^. Many studies suggested that plasma membrane intrinsic protein (PIP) aquaporins are involved in regulation of root hydraulic conductance (*L*_p_) under both osmotic and hydrostatic forces and therefore regulate whole root water uptake^9–11^. Under salt stress conditions, regulation of root water uptake is more crucial to overcome stress injury than that of leaf transpiration. The rate of root water uptake is ultimately regulated by aquaporin activity and, to some extent, by suberin deposition^8^. A decrease in *L*_p_ under saline conditions has frequently been observed and the initial decrease in *L*_p_ upon salt exposure was correlated with a down-regulation of *PIP* genes^12–15^. The decrease in *L*_p_ under salt stress might be a strategy to diminish water flow from roots to soil while the soil osmotic potential is lower than that of the roots^8^. After few days of salt stress, a partial or total recovery of *L*_p_ alonged with accumulation of PIP proteins in roots has been reported in some species, which should be accompanied by an osmotic adjustment of the root cells in order to avoid cell dehydration^12, 15–17^.

Phytohormones play critical roles in regulating plant responses to stress^18^. The contents of abscisic acid (ABA), ethylene (ET), jasmonic acid (JA), and cytokinins (CKs) as well as enzymes related to their biosynthesis exhibited significant changes under salt stress^18, 19^. ABA is an important internal signal which can be induced by salt stress^20^. Many ABA responsive transcription factors can be induced by ABA to promote expression of salt tolerant genes and therefore increase salt tolerance of plants^18^. It is well known that ABA can increase root hydraulic properties by increasing *PIP* expression and protein abundance^21–23^. In most cases, ABA is correlated with the water potential of leaf or soil, suggesting that salinity-induced increase in endogenous ABA is due to water deficit rather than specific salt effect^24^. Like ABA and ET, JA biosynthesis have also been enhanced in plant under salt stress and these activate many vital processes to cope with stress^25–28^. On the contrary, salt stress decreased the expression of isopentenyltransferases (*IPT*) genes *SlIPT3* and *SlIPT4* in Tomato (*Solanum lycopersicum* L.) and overexpression of *SlIPT3* increased salt tolerance of transgenic tomato^19^.

Plant response to salt stress varies greatly with soil environmental conditions^29, 30^. Soil salinity is often heterogeneous in saline fields, and many studies have shown that crops grow better in heterogeneous (non-uniform salinity) conditions than in uniform salinity conditions^29–34^. Non-uniform salinity has been simulated with a split-root system in a greenhouse or growth chamber, in which the root system was divided into two or more equal portions and each portion irrigated with varied concentrations of NaCl solution^30, 31^. Non-uniform salinity alleviated plant salt damage by decreasing Na^+^ concentration and osmotic stress in leaf, and increasing water and nutrient uptake by roots in the low-saline side and enhancing Na^+^ efflux from the low salinity root side via SOS1^33–36^. However, the underlying molecular mechanism of the increased water and nutrient uptake in the low-salinity root side leading to alleviation of salt damage is far from clear.

In the present study, using a split-root system to simulate non-uniform root zone salinity, we performed RNA-Seq on leaf and root samples of cotton plants under uniform- and non-uniform salinity treatments, and analyzed the global changes in the leaf and root of different treatments. The objectives were to investigate, (i) the mechanism of the improved plant growth and decreased leaf Na^+^ content under non-uniform salinity by analyzing the expression patterns of the sodium transport and photosynthesis related genes in leaves; (ii) the mechanism of increased water and nutrient uptake by roots in the non-saline root side by analyzing the expression patterns of the water and nutrient uptake related genes in roots and (iii) the expression patterns of the hormone related genes and transcription factor genes in the roots.

## Results

### RNA-Seq analysis and identification of differentially expressed genes

RNA-Seq analysis was performed on the leaves and roots of NaCl-free, uniform salinity and non-uniform salinity treatments at 6 h after salt stress (HAS). We generated more than 9.1 million raw tags in each library. After filtering out the low quality tags, we obtained clean tags ranging from 8.8 to 11.6 million per library (SRA submission number: SRP068502). The gene sequences of *G*. *hirsutum* genome were used as reference to align and identify the sequencing reads. This allowed for the mapping of approximately 80% of the distinct clean tags that passed our filters, representing more than 7.1 million reads per library with about 94% of them mapped unique reference genes (Supplemental Table S1).

**Figure 1:**
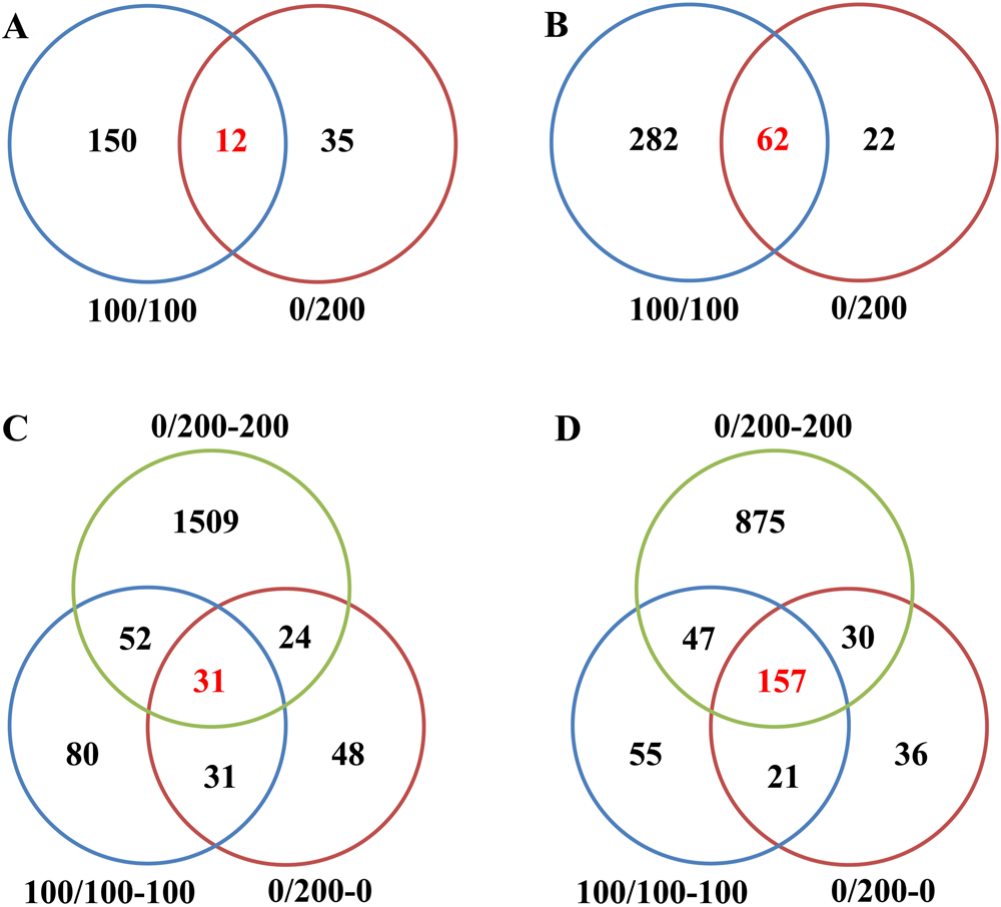
Venn diagram of genes identified as up- (**A**) and down- (**B**) regulated in leaves and up- (**C**) and down- (**D**) regulated in roots under uniform and non-uniform salinity treatment.

Putative differentially expressed genes were finally selected depending on the expression profiles and whether: (a) the average fold change between two treatment genes was more than or equal to two folds, and (b) the false discovery rate (FDR) was less than 0.001. Accordingly, 506 differentially expressed genes (DEGs) were identified in leaves under uniform salinity treatment, whereas only 131 DEGs were identified in non-uniform salinity treatment compared with NaCl-free control (Fig. 1A,B). There were 12 common up-regulated genes and 62 common down-regulated genes in leaves of uniform and non-uniform salinity treatments (Fig. 1A,B). 474, 378 and 2725 DEGs were identified in roots under uniform salinity, non- and high-saline root sides (Fig. 1C,D).

### Functional classification of differentially expressed genes in leaves

Gene ontology (GO) analysis was performed by mapping each DEG into the records of the GO database (http://www.geneontology.org/). The GO annotation of DEGs in leaf showed that 24 groups, such as response to salt stress, chlorophyll catabolic process and response to ABA stimulus etc, were identified in uniform salinity treatment, whereas only 15 groups like photosynthesis, response to salt stress and ABA stimulus, etc. were identified in non-uniform salinity treatment (Supplemental Fig. S1). As shown in Supplemental Fig. S1, many groups related to response to oxidative stress, response to salt stress and response to ABA stimulus were identified in both non-uniform and uniform salinity treatments and these gene expression patterns were similar, though the gene numbers in each group under non-uniform treatment was lower than that under uniform salinity treatment. Interestingly, 3 DEGs related to photosynthesis and light harvesting were all up-regulated under non-uniform treatment, but most of genes related to catabolic process of light-harvesting complex II and chlorophyll were up-regulated under uniform treatment (Supplemental Fig. S1).

### Expression analysis of some important genes and Pn rate and ion contents in leaves under non-uniform and uniform salinity treatments

**Figure 2:**
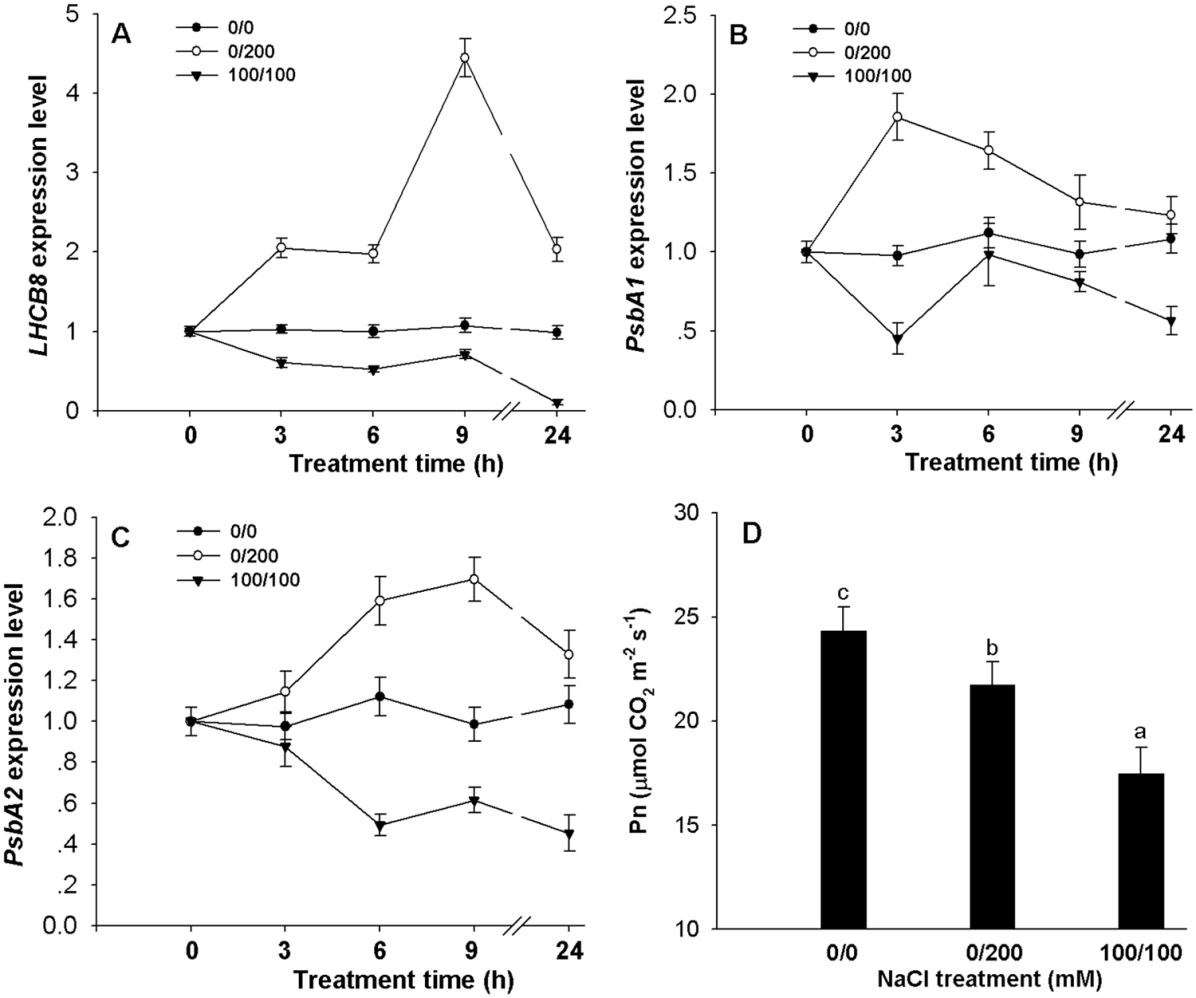
The expression patterns of *Lhcb8* (**A**), *PsbA1* (**B**) and *PsbA2* (**C**) in main-stem leaves under uniform (100/100 mM NaCl) and non-uniform (0/200 mM NaCl) salinity treatments and net photosynthetic (Pn) in leaves at 1 day after salinity treatments. Data are means of six biological replicates (±SD). Different letters indicate a significant difference (*P* < 0.05) within each panel.

Twenty five DEGs were up-regulated under non-uniform treatment but down-regulated under uniform treatment (Table 1). The expression of the 3 photosynthesis-elated genes, *Lhcb8*, *PsbA1* and *PsbA2* increased in the leaves under non-uniform salinity but decreased in those under uniform salinity (Fig. 2A–C). The leaf Pn under non-uniform salinity was higher than under uniform salinity at 1 day after treatment (DAT) although the Pn significantly decreased under both uniform and non-uniform salinity (Fig. 2D).

**Table 1:** Summary of differentially expressed genes in leaves which were significantly up-regulated in non-uniform treatment but down-regulated in uniform treatment or vice-versa.

Gene ID (Cotton_D_gene_)	Log_2_[(100/100)/(0/0)]	Log_2_[(0/200)/(0/0)]	Gene annotation
10022083	−2.383	1.220	Fasciclin-like arabinogalactan protein 19 [*Gossypium hirsutum*]
10014653	−1.620	1.488	Fasciclin-like arabinogalactan protein 19 [*Gossypium hirsutum*]
10031440	−1.072	1.174	GDSL-like Lipase/Acylhydrolase superfamily protein [*Theobroma cacao*]
10031813	−0.986	1.067	GDSL-like Lipase/Acylhydrolase superfamily protein [*Theobroma cacao*]
10008000	−2.239	1.306	Li-tolerant lipase 1 isoform 1 [*Theobroma cacao*]
10015446	−1.824	0.774	Cu-predoxin superfamily protein [*Theobroma cacao*]
10010133	−1.669	1.509	Proline-rich protein [*Gossypium hirsutum*]
10005614	−1.557	1.737	HCO_3_-transporter family isoform 1 [*Theobroma cacao*]
10033947	−1.023	1.429	SKU5 similar 5 isoform 1 [*Theobroma cacao*]
10023571	−1.375	1.142	SKU5 similar 5 isoform 1 [*Theobroma cacao*]
10010899	−1.683	0.818	Predicted protein [*Populus trichocarpa*]
10033759	−1.191	1.034	Uncharacterized protein TCM_029927 [*Theobroma cacao*]
10040515	−1.314	1.047	Uncharacterized protein TCM_000740 [*Theobroma cacao*]
10021939	−1.127	1.137	Polygalacturonase 2 [*Theobroma cacao*]
10001543	−1.297	0.769	Beta-tubulin 1 [*Gossypium hirsutum*]
10032154	−1.139	0.708	Beta-tubulin 2 [*Gossypium hirsutum*]
10039398	−1.112	0.901	Alpha-tubulin [*Gossypium hirsutum*]
10001239	−0.479	1.003	Light-harvesting complex II protein Lhcb8 [*Theobroma cacao*]
10014103	−0.752	1.653	PsbA1 [*Cardiandra alternifolia*]
10015895	−0.578	1.471	PsbA2 [*Dianthus versicolor*]
10021012	−0.639	1.853	Xyloglucan endotransglucosylase/hydrolase 16 [*Theobroma cacao*]
10022459	−0.552	1.332	Xyloglucan endotransglucosylase/hydrolase [*Gossypium hirsutum*]
10001483	−1.019	0.678	Xyloglucan endotransglucosylase/hydrolase [*Gossypium hirsutum*]
10025800	−1.5	0.671	Cytochrome P450, putative [*Theobroma cacao*]
10036871	−2.854	2.819	Gibberellin-regulated family protein, putative [*Theobroma cacao*]

**Figure 3:**
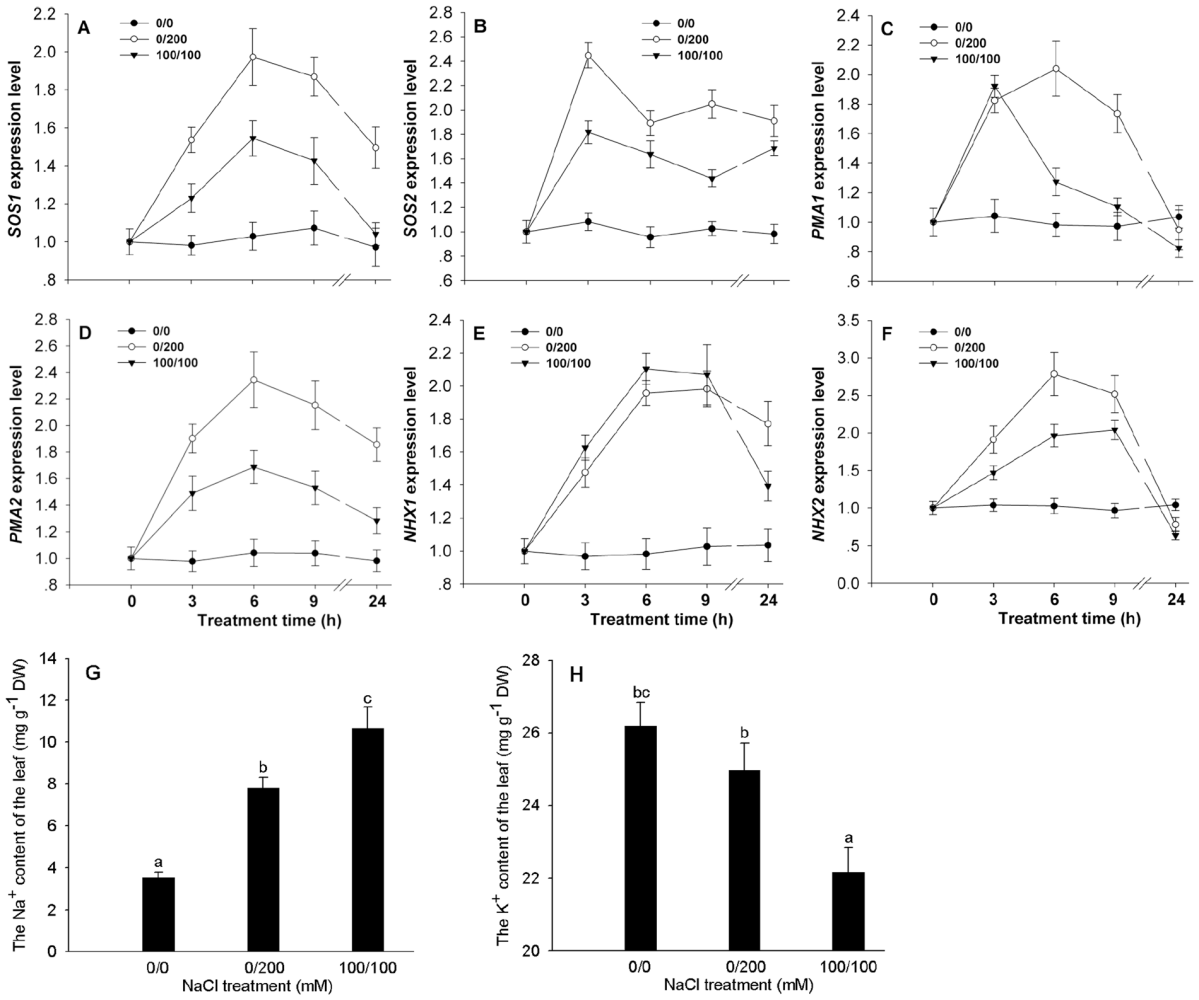
The expression patterns of *SOS1* (**A**), *SOS2* (**B**), *PMA1* (**C**), *PMA2* (**D**), *NHX1* (**E**) and *NHX2* (**F**) in leaves under uniform (100/100 mM NaCl) and non-uniform (0/200 mM NaCl) salinity treatments and Na^+^ (**G**) and K^+^ (**H**) contents in leaves at 1 day after salinity treatments. Data are means of six biological replicates (±SD). Different letters indicate a significant difference (*P* < 0.05) within each panel.

To determine if sodium transport related genes in leaves of the non-uniform and uniform salinity treatments were up-regulated as described in RNA-Seq data and check their temporal expression patterns, the expression patterns of *SOS1*, *SOS2*, plasma membrane H^+^ ATPase (*PMA1*, *PMA2*), *NHX1* and *NHX6* were analyzed by real-time PCR at 3, 6, 9 and 24 HAS. The expression of these genes in both non-uniform and uniform leaves increased gradually after salt stress and most of them peaked at 6 HAS (Fig. 3). The expression of *SOS1*, *SOS2*, *PMA2* and *NHX2* in the non-uniform leaf was higher than that in the uniform leaf after salt stress (Fig. 3A,B,D and F). The expression of *PMA1* in the non-uniform leaf was higher than that in the uniform leaf at 6, 9 and 24 HAS (Fig. 3C). The expression of *NHX1* in the non-uniform leaf was higher than that in the uniform leaf at 24 HAS (Fig. 3E). The leaf Na^+^ content under non-uniform salinity was significantly lower than that under uniform salinity though salt stress increased the Na^+^ content under both uniform and non-uniform salinity at 1 DAT (Fig. 3G). In contrast, the leaf K^+^ content under non-uniform salinity was significantly higher than that under uniform salinity (Fig. 3H).

### Functional classification of differentially expressed genes in root

**Figure 4:**
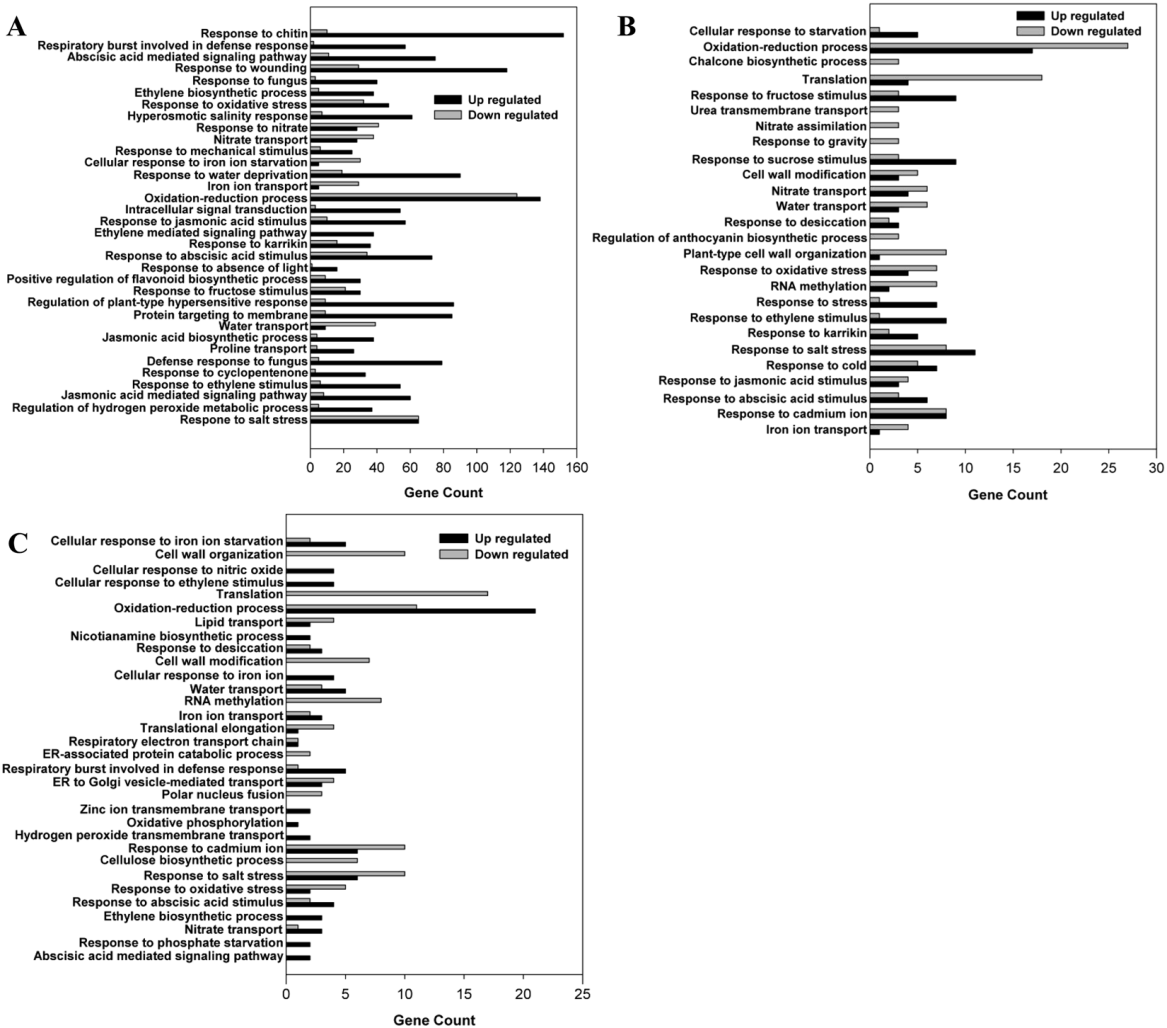
GO analysis of differentially expressed genes in roots of the high-saline root side (0/200-200) (**A**), uniform-salinity treatment (100/100-100) (**B**) and non-saline root side (0/200-0) (**C**) obtained from RNA sequencing. The abscissa of the bar plot represents the gene count within each GO category. All processes listed had enrichment *p* values < 0.05.

The GO annotation of DEGs in root is presented in Fig. 4. The main functional groups related to salt stress, oxidative stress, water deprivation, etc. were up-regulated in high-saline root side of non-uniform salinity treatment (Fig. 4A). Most of the genes related to ABA, ET and JA mediated signaling pathway and response to ABA, ET and JA stimulus and ethylene biosynthetic process were also up-regulated in the high-saline root side of the non-uniform salinity treatment (Fig. 4A). However, the main functional groups of down-regulated genes were related to response to nitrate, nitrate transport, cellular response to iron ion starvation, iron ion transport and water transport (Fig. 4A). Many up-regulated genes in the high-saline root side, such as response to ABA and ET stimulus, were still up-regulated in the uniform salinity root and non-saline root side (Fig. 4).

Interestingly, most of the genes related to nitrate transport, iron transport and water transport were down-regulated in both root sides of uniform salinity treatment and the high-saline root side of non-uniform salinity treatment, but most of these were up-regulated in the non-saline root side under non-uniform salinity (Fig. 4). Surprisingly, the DEGs related to cellular responses to nitric oxide, ET stimulus, iron, zinc ion transmembrane transport, oxidative phosphorylation, hydrogen peroxide transmembrane transport, ET biosynthetic process, response to phosphate starvation and ABA mediated signaling pathway were all up-regulated in the non-saline root side (Fig. 4C).

### Differential expression of nutrient transport genes and nutrient uptake in roots under non-uniform and uniform salinity treatments

Analysis of expression level of nutrient transport genes in roots of uniform and non-uniform salinity showed 13 nitrate, 7 potassium and 10 phosphate transport-related genes in the RNA-Seq data. Ten of the 13 nitrate transport-related genes were down-regulated in the high-saline root side and uniform salinity root (Table 2). Interestingly, 7 nitrate transport-related genes which were down-regulated in the high-saline root side were up-regulated in the non-saline side root except the other 3 up-regulated nitrate transport-related genes in all treatment roots (Table 2). The 7 potassium transport-related genes were all down-regulated in the high-saline root side and uniform salinity root and the expression of these genes were all lower than that in non-saline root side (Table 2). There were 10 phosphate transport-related genes which were up-regulated in all treatment roots (Table 2).

**Table 2:** The expression pattern of nitrate, potassium and phosphate transport genes in roots under uniform- and non-uniform salinity treatments.

Nutrition	Gene ID (Cotton_D_gene_)	Log_2_ [(100/100-100)/(0/0-0)]	Log_2_ [(0/200-0)/(0/0-0)]	Log_2_ [(0/200-200)/(0/0-0)]	Gene annotation
Nitrate	10000609	−1.748128	0.430718	−2.86098	Nitrate transporter 1.1 [*Theobroma cacao*]
	10006958	−1.808451	−0.6115	−4.71918	Nitrate transporter 2:1 [*Theobroma cacao*]
	10008863	1.1008006	0.291231	3.14439	Nitrate transporter 1:2 [*Theobroma cacao*]
	10009751	−1.359804	0.25331	−4.77721	Nitrate transporter 1.1 [*Theobroma cacao*]
	10009753	−0.933707	0.737786	−2.98314	Nitrate transporter 1.1 [*Theobroma cacao*]
	10014248	−0.156504	0.114736	−2.19127	Nitrate transporter 1.7 [*Theobroma cacao*]
	10019505	−0.6206	0.235432	−2.13213	Nitrate transporter [*Arabidopsis thaliana*]
	10024700	1.1300605	0.442005	3.169925	Nitrate transporter 1.5 [*Theobroma cacao*]
	10032251	−1.007627	−0.4728	−2.90436	Nitrate excretion transporter 1 [*Theobroma cacao*]
	10032252	−1.430634	−0.47038	−3.09622	Nitrate excretion transporter 1 [*Theobroma cacao*]
	10033454	1.4188291	0.074001	0.185032	Nitrate transporter 1:2 [*Theobroma cacao*]
	10022762	−0.269833	0.468746	−1.6487	Nitrate transporters [*Theobroma cacao*]
	10037760	−0.859288	0.073784	−6.15915	Nitrate transporter 1.5 [*Theobroma cacao*]
Potassium	10008417	−0.1256	0.23249	−1.2006	Potassium uptake transporter 3 [*Theobroma cacao*]
	10016252	−0.75473	−0.60145	−2.31438	Potassium transporter 2 [*Theobroma cacao*]
	10016708	−0.520023	0.055607	−2.1062	Potassium transporter 2 [*Theobroma cacao*]
	10018786	−0.295198	0.095238	−2.91544	Potassium transporter [*Theobroma cacao*]
	10027906	−1.236198	−0.42094	−1.87055	Potassium transporter family protein [*Theobroma cacao*]
	10033349	−0.321	0.7571	−2.5697	High affinity K + transporter 5 [*Theobroma cacao*]
	10026743	−0.573044	0.099487	−1.38229	Potassium channel in 3 [*Theobroma cacao*]
Phosphate	10022858	4.9166667	1.916667	13.9	Phosphate transporter 3,1 [*Theobroma cacao*]
	10021985	0.7352941	1.303922	0.235294	Phosphate transporter 2,1 [*Theobroma cacao*]
	10021898	1.1921397	0.633188	1.864629	Phosphate transporter 1,4 [*Theobroma cacao*]
	10024110	0.7725118	0.808057	0.279621	Phosphate transporter 4,3 [*Theobroma cacao*]
	10010804	0.4825397	0.76	0.777778	Phosphate transporter 3,1 [*Theobroma cacao*]
	10036742	0.7368421	0.763158	0.052632	Phosphate transporter 1,9 [*Theobroma cacao*]
	10002982	−0.1568	0.1263	−2.83636	Phosphate transporter 1,7 [*Theobroma cacao*]
	10014884	1.2432432	0.297297	22.27027	EXS family protein [*Theobroma cacao*]
	10022222	0.5428571	0.819048	0.2	EXS family protein [*Theobroma cacao*]
	10040038	0.677792	0.782413	0.273427	Phosphate 1 [*Theobroma cacao*]

**Figure 5:**
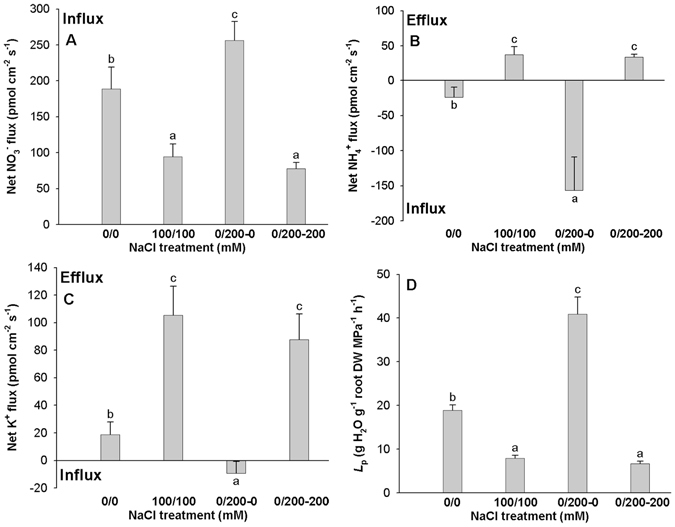
Effects of non-uniform (0/200 mM) and uniform (0/0 and 100/100) root zone salinity on net NO_3_^−^ (**A**), NH_4_^+^ (**B**) and K^+^ (**C**) fluxes and Hydraulic conductance (*L*_p_) (**D**) in roots of cotton at 6 h after treatment. The data are main fluxes of NO_3_^−^, NH_4_^+^ and K^+^ within the measuring periods (15 min). Data are means of six biological replicates (±SD). Bars with different letters (a, b, and c) differ significantly at *P* < 0.05.

A net NO_3_^−^ influx was observed in cotton roots under both NaCl-free and salt stress conditions, but the net influx in the high-saline root side and the uniform salinity root were significantly lower than that in the NaCl-free control (Fig. 5A). However, the net NO_3_^−^ influx in the non-saline root side was significantly higher than that in the NaCl-free control (Fig. 5A). A net NH_4_^+^ influx in roots of the NaCl-free and non-saline root side was also observed but the net influx in the non-saline side was higher than that in the NaCl-free control (Fig. 5B). The net NH_4_^+^ flux were reversed to efflux in either root side under uniform salinity and in the high-saline root side under non-uniform salinity (Fig. 5B). A net K^+^ influx was observed in the non-saline root side under non-uniform salinity, but net K^+^ flux under the NaCl-free treatment, in either root side under uniform salinity, and the high-saline root side under non-uniform salinity was reversed to efflux (Fig. 5C).

### Differentially expressed aquaporin genes and *L*_p_ in roots under non-uniform and uniform salinity treatments

There were 27 differentially expressed aquaporin genes, of which 24 were down-regulated in the high-saline root side and 21 down-regulated in the uniform salinity root compared with NaCl-free control (Table 3). Unlike the high-saline root side, most of the aquaporin genes (18) were up-regulated in the non-saline root side (Table 3). The expression levels of the 24 genes down-regulated in the high-saline root side were higher in the non-saline root side than that in the high-saline and uniform salinity roots, with 15 of the genes up-regulated in the non-saline root side (Table 3). The 3 up-regulated genes in the high-saline root side occurred in the non-saline root side and uniform salinity roots (Table 3). Consistent with the decreased expression of aquaporin genes, the *L*_p_ in either root side of the uniform salinity and high-saline root side of the non-uniform salinity treatment also decreased, but the *L*_p_ of the non-saline root side under non-uniform salinity increased by 116.4% compared with NaCl-free control (Fig. 5D).

**Table 3:** The expression patterns of aquaporin genes in roots under uniform and non-uniform salinity treatments, which were significantly up- or down-regulated in the high-saline root sides.

Gene ID (Cotton_D_gene_)	Log_2_ [(100/100-100)/(0/0-0)]	Log_2_ [(0/200-0)/(0/0-0)]	Log_2_ [(0/200-200)/(0/0-0)]	Gene annotation
10009326	−0.33267	0.424425	−2.42742	PIP protein [*Gossypium hirsutum*]
10004201	−0.39015	0.379398	−3.7498	TPA: TPA_inf: aquaporin TIP1;4 [*Gossypium hirsutum*]
10018179	−2.21045	−0.06931	−1.42916	PIP protein [*Gossypium hirsutum*]
10006595	−0.07887	1.016425	−2.24595	Plasma membrane intrinsic protein 2,4 [*Theobroma cacao*]
10038574	−0.76512	−0.16798	−1.7053	aquaporin PIP2;3 [*Gossypium hirsutum*]
10008252	−2.14325	0.392041	−2.23742	Tonoplast intrinsic protein 2,3 [*Theobroma cacao*]
10020312	−1.24614	−0.34652	−1.91007	aquaporin SIP1;7, partial [*Gossypium hirsutum*]
10019562	−2.07288	−0.73534	−1.05041	Plasma membrane intrinsic protein 2A [*Theobroma cacao*]
10015612	−0.30775	−0.19935	−1.13458	aquaporin TIP2;3 [*Gossypium hirsutum*]
10022627	−0.35396	0.049896	−2.02539	NOD26-like intrinsic protein 5,1 [*Theobroma cacao*]
10032274	−0.25704	0.23041	−1.83799	Plasma membrane intrinsic protein 2,4 [*Theobroma cacao*]
10009738	0.610744	0.871895	−1.49557	Tonoplast intrinsic protein 2,3 [*Theobroma cacao*]
10032221	−0.0926	0.802992	−3.08491	Tonoplast intrinsic protein 1,3 [*Theobroma cacao*]
10004979	−0.7341	0.819265	−2.10376	Tonoplast intrinsic protein 1,3 [*Theobroma cacao*]
10036429	−2.88826	−0.65004	−2.97082	TPA: TPA_inf: aquaporin TIP4;1 [*Gossypium hirsutum*]
10024645	0.937983	0.460841	2.726458	PIP1 protein [*Gossypium hirsutum*]
10009325	0.219162	0.54999	−2.02131	PIP protein [*Gossypium hirsutum*]
10025289	−1.62286	−0.05523	−3.17146	aquaporin TIP1;7 [*Gossypium hirsutum*]
10004995	−1.77047	−0.45767	−1.64657	TPA: TPA_inf: aquaporin TIP1;5 [*Gossypium hirsutum*]
10001242	−0.27999	0.224264	−1.46605	aquaporin PIP1;11 [*Gossypium hirsutum*]
10009324	−0.28816	0.216449	−1.90118	PIP protein [*Gossypium hirsutum*]
10001728	−1.49766	−0.37941	−1.93193	tonoplast intrinsic protein [*Gossypium hirsutum*]
10014030	−0.36778	1.063372	−2.44511	Plasma membrane intrinsic protein 2,4 [*Theobroma cacao*]
10024472	−0.67203	0.873597	−2.03637	putative plasma membrane intrinsic protein PIP family member 1 aquaporin [*Pachira quinata*]
10006930	−0.6799	0.72487	−1.47373	PIP protein [*Gossypium hirsutum*]
10031445	0.934751	0.169815	2.755901	TPA: TPA_inf: aquaporin NIP1;1 [*Gossypium hirsutum*]
10001135	0.356561	0.381873	1.700648	Aquaporin sip2.1, putative [*Theobroma cacao*]

### Differentially expressed hormone related and transcription factor genes in roots

Four *IPT* genes were significantly down-regulated in the high-saline root side and uniform salinity root and their expression was lower than that in the non-saline root side (Supplemental Table S2). Four 9-cis-epoxycarotenoid dioxygenase (*NCED*) genes were up-regulated in roots of uniform and non-uniform salinity treatment, and their expression was higher than that in the non-saline root side (Supplemental Table S2). In contrast, the other 3 ABA biosynthesis aldehyde oxidase (*AAO*) genes were all down-regulated in all uniform and non-uniform salinity roots and their expression in the high-saline root side and uniform salinity root was lower than in the non-saline root side (Supplemental Table S2). Surprisingly, the expression of 4 ABA catabolic genes *CYP707A* in the high-saline root side and uniform salinity root were increased, being higher than in the non-saline root side, although 2 *CYP707A* genes were also up-regulated in the non-saline root side. As for the 5 differentially expressed ethylene biosynthesis genes ACC oxidase (*ACO*), 4 of them were up-regulated in all uniform and non-uniform salinity roots and their expressions in the high-saline root side were higher than in non-saline root side (Supplemental Table S2).

There are a large number of transcription factors (TFs) in plants to perceive and mediate responses to environmental changes which act as the earliest and vital players during stresses. We found 47, 16 and 144 up-regulated TFs and 41, 6 and 105 down-regulated TFs in roots under uniform salinity, non- and high-saline root side (Supplemental Table S3). The expression pattern of the differentially expressed NAC, WRKY, GRAS, MYB and Nuclear Y subunit TFs in the non-saline root side were similar to that in the high-saline root side and uniform salinity root (Supplemental Table S4). There are only 4 ERF TFs which have similar expression pattern in the non- and high-saline root sides though 9 differentially expressed ERF TFs were found (Supplemental Table S4).

### Confirmation of Solexa Expression Patterns by RT-PCR Analysis

To validate the results of the gene expression analysis obtained by RNA-Seq, RT-PCR analysis was performed for a subset of 9 genes in leaf and 11 genes in root as identified by RNA-Seq. The results showed that 48 of the 51 gene expression data had similar expression profiles as the original RNA-Seq (Supplemental Table S5). This indicates that the original data of RNA-seq was validated in 94.1% of the cases. This was not the case for the other gene presumably because the RNA used for RNA-seq and RT-PCR was extracted from different plants. The expression patterns of the 20 genes were highly consistent with the RNA-seq ratios, with a relative R^2^ of 0.8215 (Supplemental Fig. S2, Supplemental Table S5).

## Discussion

Salt stress caused ion toxicity, osmotic stress and nutrient deficiency and thus affected plant growth by up- or down-regulating many salt-related genes in cotton^4, 5, 37, 38^. In our study, 506 DEGs were identified in the leaf under uniform salinity, whereas only 131 DEGs were identified under non-uniform salinity. The results suggested that plants under non-uniform salinity suffer less salt stress than those under uniform salinity. Salt stress decreased leaf photosynthesis, and many genes involved in the photosynthesis pathway were down-regulated^34, 37–39^. It was reported that total energy gain and plant growth decreased with greater salinity stress by decreasing photosynthetic rate following induced damage to cellular and photosynthetic machinery^40^. Our data showed that many genes involved in photosynthesis were down-regulated in leaves under both uniform and non-uniform salinity treatments, but the number of down-regulated genes under non-uniform treatment was lower than that under uniform treatment. These results may explain the increased plant growth under non-uniform. Most of chlorophyll and light-harvesting complex II catabolic process related genes which have negative function on photosynthesis were up-regulated in leaves under uniform salinity treatment. However, the positive genes *Lhcb8*, *PsbA1* and *PsbA2* on photosynthesis were up-regulated in the non-uniform salinity treatment, which may be the reason for the increased Pn relative to the uniform salinity treatment (Fig. 2; Table 1).

Salt stress induced Fasciclin-like arabinogalactan, Li-tolerant lipase, Xyloglucan endotransglucosylase/hydrolase and Cytochrome P450 genes play important roles in plant salt tolerance. Their overexpression increased the salt tolerance of transgenic plants^41–45^. In this study, 25 down-regulated genes under uniform salinity treatment, which included the genes mentioned above, were up-regulated under non-uniform salinity. The increased expression of these genes may contribute to the increased salt tolerance and hence decreased salt damage under the non-uniform salinity (Table 1).

Maintaining ionic homeostasis is critical for plant to cope with saline environments. SOS pathway proteins and H^+^-ATPase can be induced to transport Na^+^ out of the cytoplasm while NHXs can also be induced to sequester Na^+^ in the vacuole to reduce ionic toxicity in plant leaves under salt stress^4, 7, 46–49^. The expression of *SOS1*, *SOS2*, *PMA1*, *PMA2*, *NHX1* and *NHX6* genes in leaves were all up-regulated under salt stress and the expression of most of these genes was higher under non-uniform than uniform salinity. This may be an important reason for the reduced leaf Na^+^ content and salt damage under non-uniform salinity (Fig. 3). The high expression of these sodium related genes in the non-uniform salinity may be ascribed to some signals originating from the high-saline root side, implying that the high-saline root can induce some important salt tolerant genes to increase the salt tolerance of cotton.

Roots play a primary role in particular changes occurring in plants because they are directly in contact with the soil and absorb water and other essential nutrients from the soil^18, 50^. Root systems have important roles in improving crop salt tolerance through increasing water and nutrients uptake and limiting salt acquisition, although salt stress limits water and nutrient uptake by roots^51^. Aquaporin proteins, which regulate a large proportion of water transport across membranes, are rapidly influenced both transcriptionally and post-translationally by salt^52^. Moreover, many studies have shown that the uptake of water by roots is mainly mediated by PIPs^53–55^. Fetter *et al*.^56^ found that co-expression of PIP1s and PIP2s in *Xenopus laevis* oocytes led to an increase in the osmotic water permeability coefficient (Pf) and the increased Pf was attributable to the formation of tetramers by PIP1 and PIP2 proteins. In the present study, most water transport related genes were down-regulated in uniform-salinity root and the high-saline root side but most of these were up-regulated in non-saline root side, which was parallel to our previous study that water uptake decreased from the uniform- and high-saline root side but increased in the non-saline root side^34^ (Fig. 4). As shown in Table 3, 24 of the 27 differentially expressed aquaporin genes in the high-saline root side were down-regulated, whereas most of these were up-regulated in the non-saline root side. The root *L*_p_ under uniform salinity and high-saline side decreased but that in the non-saline side increased (Fig. 5D). These results suggested that the increased water uptake may be due to the increased *L*_p_ as measured by increased expression of aquaporin genes in the non-saline root side. The increased water uptake in the non-saline root side may decrease osmotic stress and then alleviate salt damage under non-uniform salinity.

Plant growth can be adversely affected by salinity-induced nutrient imbalance through changes in nutrient availability, competitive uptake, transport or partitioning within the plant^57^. Nutrient uptake by active transport through the roots is the first major step to enhance nutrient use in any plant. Many studies have shown that salinity can directly affect nutrient uptake, such as reducing N, P and K uptake and decreasing the expression of high affinity nitrate transporters, *AtNRT2*.*1* and *AtNRT2*.*2* ^4, 57–59^. Our previous study has shown that the non-saline root side uptakes more nutrients than the high-saline root side under non-uniform salinity^36^. In this study, most of the differentially expressed genes related to nitrate, potassium and phosphate transport were up-regulated in the non-saline root side, but most were down-regulated in the high-saline root side and uniform salinity treatment (Fig. 4; Table 2). The net NO_3_^−^ influx in the non-saline side root was significantly higher than in the high-saline root side under non-uniform salinity and in either root side under uniform salinity. Similarly, the net NH_4_^+^ and K^+^ influx in roots of the non-saline root side were higher than in the high-saline side and the uniform salinity root because the net NH_4_^+^ and K^+^ flux were reversed to efflux in the uniform salinity root and high-saline side root after salt stress (Fig. 5A–C). The increased NO_3_^−^, NH_4_^+^ and K^+^ influx in the non-saline side root may be due to the increased expression of nutrient transport related genes, which possibly contributed to the increased nutrient uptake in the non-saline root side under non-uniform salinity. The increased nutrient uptake in the non-saline root side under non-uniform salinity mitigated nutrient deficiency and thus alleviated salinity damage.

It is well known that ABA modifies root hydraulic properties by increasing *L*_p_, PIP aquaporin expression and protein abundance^21–23^. The increased expression of *NCED* genes and decreased expression of ABA catabolic genes *CYP707A* may increase ABA content in the non-saline root side, which may be used as an important positive signal to increase PIP aquaporin expression and then increase water uptake from the non-saline root side (Supplemental Table S2).

Transcription factors are known to play vital roles in abiotic stress signaling in plants. Genome-wide transcriptome analysis revealed that a number of TFs were induced or repressed in response to abiotic stresses in cotton^38, 60–62^. In this study, 144 up-regulated and 105 down-regulated TFs were identified in the high-saline root side and 16 up-regulated and 5 down-regulated TFs were identified in the non-saline root side. Most of the TFs in NAC, ERF and WRKY families were up-regulated in the high-saline root side and 6 NAC, 4 ERF and 1 WRKY genes were induced in the non-saline root side. The results suggested that these genes may play important roles in cotton salt tolerance as reported previously^63^.

Plant roots are the first tissues to encounter salt or other osmotic stresses. Alterations in root hormones like CK, ABA, ET and IAA, could mediate root-to-shoot signaling in regulating shoot growth and physiology, and ultimately agricultural productivity^64, 65^. Root-specific induction of *IPT* gene could enhance root-to shoot cytokinin signaling, and thus delayed leaf senescence and improved plant growth^64, 65^. Plant root plasticity to fluctuating environments is a primary mechanism for optimizing water and nutrient acquisition through enhanced uptake/assimilation systems, and proliferation specifically in nutrient-rich zones depends on the integration of local and systemic signaling^66^. Studies in systemic N signaling using a split-root system showed that root growth under low N condition is controlled by the signal from the shoot^66^. Recently, Suzuki *et al*.^67^ reported that temporal–spatial interaction between reactive oxygen species (ROS) and ABA could regulate rapid SAA to heat stress in plants. These results suggests that systemic acquired acclimation (SAA) plays a key role in plant survival during stress. In our study, the increased expression of water and nutrient transport and hormone related genes, such as *IPT*, *NCED* and *ACC* in the non-saline root side may serve as positive signals from root to enhance cotton growth under the non-uniform salinity treatment. On the one hand, the enhanced expression of sodium related genes in the shoot may be induced by the high-saline root side; On the other hand, the increased expression of water and nutrient transport genes, TF and hormone related genes may be induced by some signals from the shoot. It seemed that both root-to-shoot and shoot-to-root signals were required to explain the decreased salt damage under non-uniform salinity.

## Materials and Methods

### Plant material preparation and salinity treatment

A commercial cotton (*Gossypium hirsutum* L.) cultivar, K836 developed by the Cotton Research Center, Shandong Academy of Agricultural Sciences, Jinan, was used in the experiment. Acid-delinted seeds were sown at ~3 cm depth in plastic boxes (60 cm × 45 cm × 15 cm) containing sterilized wet sand. The boxes were placed in growth chambers with light/dark regimes of 16/8 h, light intensity of 400 μmol m^−2^ s^−1^ PAR, and temperature of 30 ± 2 °C. At full emergence, seedlings were thinned to 100 plants per box.

When most seedlings reached the two-true leaf stage, uniform seedlings were carefully removed from the sand and washed with distilled water. Split-root systems were established by grafting with these seedlings as described in Kong *et al*.^34^. Grafted seedlings were transferred to plastic pots containing aerated nutrient solution. The solution consisted of (mM): 1.25 Ca(NO_3_)_2_, 1.25 KNO_3_, 0.5 MgSO_4_, 0.25 NH_4_H_2_PO_4_, 0.05 EDTA-FeNa; and (μM): 10 H_3_BO_3_, 0.5 ZnSO_4_, 0.1 CuSO_4_, 0.5 MnSO_4_, 0.0025 (NH_4_)_6_Mo_7_O_24_, and was adjusted to pH 6 with KOH. When a new leaf emerged from the grafted seedling 2 weeks after grafting, the plastic bag and parafilm were removed. Grafted seedlings with two uniform split-root systems were transferred to the greenhouse to grow under a 14/10 h (light/dark) photoperiod at 30/26 °C and relative humidity of 60/80% for 30 d. Nutrient solutions were renewed daily during the period of growth. Healthy seedlings with uniform split roots were selected for further study.

Large plastic boxes (26 cm × 16 cm × 15 cm) were used for the uniform and non-uniform salinity treatment. Their inner space was divided into two equal parts by standing a plastic board in the middle of the boxes to produce a split-root box. The water in one side of the split-root box cannot flow into the other. Healthy seedlings with uniform roots were selected and each root portion was put into one side of the boxes. Thus the two root portions of each seedling were exposed to different NaCl concentrations at the same time. The two root portions under different salt (0 and 200 mM NaCl) treatment were denoted as non-uniform salinity treatment (0/200 mM). Treatment with the two root portions in 0 mM NaCl was denoted as NaCl-free control (0/0 mM NaCl) and treatment with the two root portions in 100 mM NaCl was denoted as uniform salinity treatment. In the non-uniform salinity treatment (0/200 mM), the NaCl-free side was considered the non-saline root side (0 or 0/200-0), while the 200 mM side was considered the high-saline root side (200 or 0/200-200); 0/0-0 and 100/100-100 denoted the root of NaCl-free and uniform salinity treatment. For each treatment, three biological replicates were designed; the samples of each biological replicate were pooled from 10 plants, the plants being randomly selected to avoid any potential effects of position within the greenhouse. After 6 h of treatment, leaves in the NaCl-free, uniform and non-uniform treatment and roots of the NaCl-free (0/0-0), uniform treatment (100/100-100), non-saline (0/200-0) and high-saline root sides (0/200-200) of the non-uniform treatment were sampled, washed 3 times with distilled water, and then frozen in liquid nitrogen and stored at −80 °C for use.

### RNA Extraction, DGE sequencing and analysis

Total RNA was extracted using the TRIzol reagent (Invitrogen), and mRNA was isolated from total RNA using Dynabeads Oligo (dT) (Invitrogen Dynal), following the manufacturer’s instructions. For RNA-Seq, total RNA from 10 representative individual plants of each treatment was mixed into one biological replicate. Approximately, 8 μg of total RNA was used. Tag libraries were prepared using the Illumina Gene Expression Sample Prep Kit, following the manufacturer’s protocol, as described in Luan *et al*.^68^. The libraries were then sequenced using an Illumina HiSeq^TM^ 2500 with 50-bp single-end (SE) reads each. The genome of *G*. *hirsutum* (ftp://ftp.ncbi.nih.gov/repository/unigene/Gossypium_hirsutum/Ghi.Seq.uniq.gz) was used as reference sequence to align and identify the sequencing reads. To map the reads to the reference, the alignments and the candidate gene identification procedure were conducted using the mapping and assembly with qualities software package^69^. Clean tags mapping to reference sequences from multiple genes were filtered out, and the remaining clean tags were designated as unambiguous clean tags. For gene expression analysis, the number of unambiguous clean tags for each gene was calculated and then normalized to TPM (number of transcripts per million clean tags)^70, 71^.

### Identification of differentially expressed genes and Functional analysis

To identify DEGs across the 3 leaf and 4 root samples, pair wise comparisons among the 3 leaf and 4 root samples were performed using a rigorous algorithm method based on a previous method^72^. The DEGs were obtained after filtering using a threshold FDR of ≤0.001 and an absolute value of log_2_ Ratio ≥2. GO enrichment analysis was performed for functional categorization of DEGs using agriGO software and the *P*-values corrected by applying the FDR correction to control falsely rejected hypotheses during GO analysis^73^. The pathway analysis was conducted using KEGG (www.genome.jp/kegg/).

To study the difference between leaves under uniform and non-uniform salinity treatments, genes down-regulated under uniform salinity but up-regulated under non-uniform salinity treatments were selected manually. Genes related to nitrate, potassium, phosphate, zinc and iron transport were also selected manually. The aquaporin genes and hormones, such as cytokinin, ABA and ethylene related genes which differentially expressed more than two folds in the non-saline (0/200-0) or high-saline root side (0/200-200) were also selected by hand. The transcription factor genes which differentially expressed in roots were also analyzed and classified.

### Real-time PCR (RT-PCR) analysis

Real-time PCR analysis was used to determine the expression of some important genes and validate the results of RNA-Seq. The samples used for RNA isolation in RT-PCR experiments were different from the samples used in RNA-Seq analysis. The samples from 15 representative individual plants of each line were harvested and every 5 samples were combined into one biological replicate and then extracted for total RNA using the TRIzol reagent (Invitrogen). cDNA fragment was synthesized from total RNA using Superscript II reverse transcriptase (Invitrogen). The specific primers for the selected genes and internal control gene (actin) are listed in Supplemental Table S6. Samples were run in triplicate on a Bio-red IQ2 Sequence Detection System and Applied Biosystems software using 0.1 mL first-strand cDNAs and SYBR Green PCR Master Mix (Applied Biosystems). The results were normalized to the expression level of actin and relative to the NaCl-free control sample. The Pearson correlation coefficients of the expression patterns of selected genes between RT-PCR and RNA-Seq were calculated using the SAS software.

### Measurement of net Pn

Net Pn of the third fully expanded young leaf from the end on the main stem were measured between 09:00 h and 11:00 h on cloudless days when ambient photosynthetic photon flux density exceeded 1500 µM m^−2^ s^−1^, using a LI-6400 portable photosynthesis system (Li-Cor, Lincoln, NE, USA).

### Measurements of net NO_3_^−^, NH_4_^+^ and K^+^ flux with NMT

Net flux of NO_3_^−^, NH_4_^+^ and K^+^ were measured by Non-Invasive Micro-Test Technology (NMT) [NMT system BIOIM; Younger USA, LLC.] as described in Kong *et al*.^34^. After exposure to the NaCl-free control and uniform and non-uniform salinity treatments for 6 h, root segments with 2–3 cm apices were sampled for ion flux measurements. Roots were rinsed with redistilled water and immediately incubated in the measuring solution to equilibrate for 30 min and then transferred to the measuring chamber containing 10–15 ml of fresh measuring solution. The measuring site was 5 mm from the root apex. The measuring solution for NO_3_^−^ and NH_4_^+^ consisted of (mM) 0.1 NH_4_NO_3_, 0.1 CaCl_2_ and 0.3 MES, adjusted to pH 6.5 with choline and HCl. The measuring solution of K^+^ consisted of (mM) 0.1 KCl, 0.1 CaCl_2_, 0.1 MgCl_2_, 0.5 NaCl, 0.3 MES and 0.2 Na_2_SO_4_, adjusted to pH 6.5 with choline and HCl. Two-dimensional ionic fluxes were calculated using MageFlux developed by Yue Xu (http://xuyue.net/mageflux). Positive values in figures represent efflux for NH_4_^+^ and K^+^ but influx for NO_3_^−^.

### Root *L*_p_

*L*_p_ of roots in both split-root compartments of the box was measured by pressurizing the roots in a pressure chamber (PMS 670, American) as described previously 24. All measurements of *L* were conducted between 11:00 and 15:00 h. The stem of each side of the split-root system was cut with a blade, 1 cm below the grafted position. The root systems were sealed into pressure chamber with the cut stump protruding through the lid and the roots surrounded by nutrient solution and chemical of the different treatments. Plastic tubes were placed in the stems to collect the exudates. Pressure was applied slowly until the root water potential was reached, and then a series of pressures applied to determine volume flows. The exuded sap was collected and weighted. Sap flow was expressed in g H_2_O g^−1^ (root dry weight) h^−1^ and plotted against pressure (MPa), with the slope being the *L* value in g H_2_O g^−1^ (root dry weight) h^−1^ MPa^−1^.

## Electronic supplementary material

Supplementary Information
