# Supplementary material for: Non-uniform salinity in the root zone alleviates salt damage by increasing sodium, water and nutrient transport genes expression in cotton
Source: Sci Rep. 2017 Jun 6;7:2879. doi: 10.1038/s41598-017-03302-x (PMC5460137; doi:10.1038/s41598-017-03302-x)
Supplement: Supplementary file 1 — Supplementary Information [file 41598_2017_3302_MOESM1_ESM.pdf]

**Title:** Non-uniform salinity in the root zone alleviates salt damage by increasing sodium, water and nutrient transport genes expression in cotton

Xiangqiang Kong, Zhen Luo, Hezhong Dong *, Weijiang Li & Yizhen Chen

Cotton Research Center, Shandong Key Lab for Cotton Culture and Physiology, Shandong Academy of Agricultural Sciences, Jinan 250100, PR of China

* To whom correspondence should be addressed. E-mail: donghz@saas.ac. cn

## Supplementary Information

Supplementary data may be found in the online version of this article.

**Fig. S1** GO analysis of differentially expressed genes in leaves under uniform salinity treatment (100/100 mM NaCl) (A) and non-uniform salinity treatment (0/200 mM NaCl) (B) obtained from RNA sequencing. The abscissa of the bar plot represents the gene count within each GO category. All processes listed had enrichment *p* values < 0.05.

**Fig. S2** Comparison of the expression ratios of some selected genes using RNA-Seq and RT-PCR.

**Supplemental Table S1** Total number of sequencing reads and unique mapped reads obtained from each sample.

**Supplemental Table S2** The expression patterns of CK, ABA and ET biosynthesis and metabolic related genes in roots under uniform and non-uniform salinity treatments, which were significantly up- or down-regulated in the high-saline root sides.

**Supplemental Table S3** Summary of differentially expressed transcription factors (TFs) in roots of uniform and non-uniform salinity treatments.

**Supplemental table S4** The expression patterns of TF genes in roots under uniform and non-uniform salinity treatments, which were significantly up- or down-regulated in the non-saline root sides.

**Supplemental table S5** The expression patterns of genes in roots and leaves under uniform and non-uniform salinity treatments, as identified by RT-PCR.

**Supplemental table S6** Primers used for RT-PCR analysis of genes described in Fig. 2 and Fig. 3.

**Supplemental table S7** Primers used for RT-PCR analysis of genes in Supplemental Table S5.

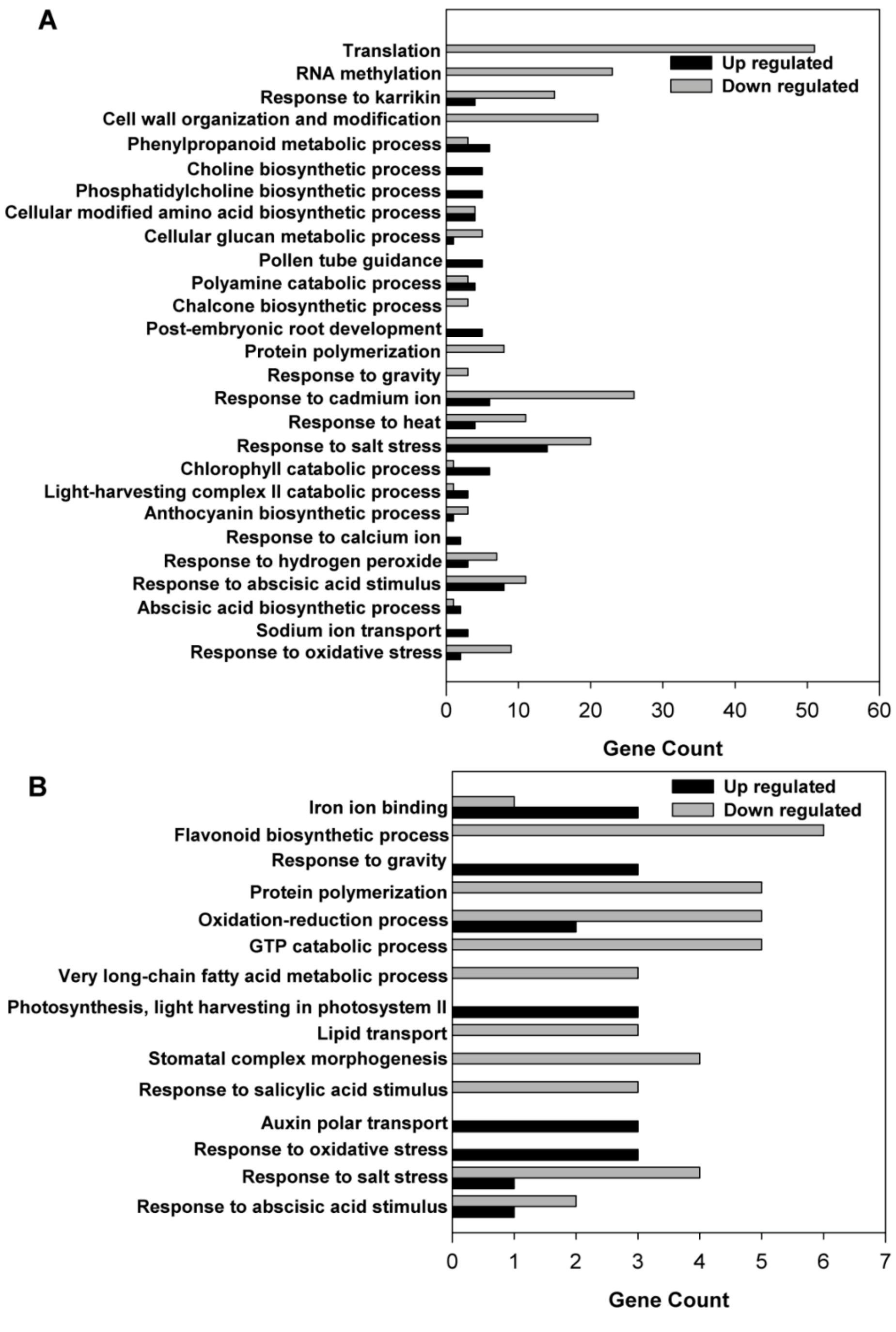


**Fig. S1** GO analysis of differentially expressed genes in leaves under uniform salinity treatment (100/100 mM NaCl) (A) and non-uniform salinity treatment (0/200 mM NaCl) (B) obtained from RNA sequencing. The abscissa of the bar plot represents the gene count within each GO category. All processes listed had enrichment $p$ values < 0.05.

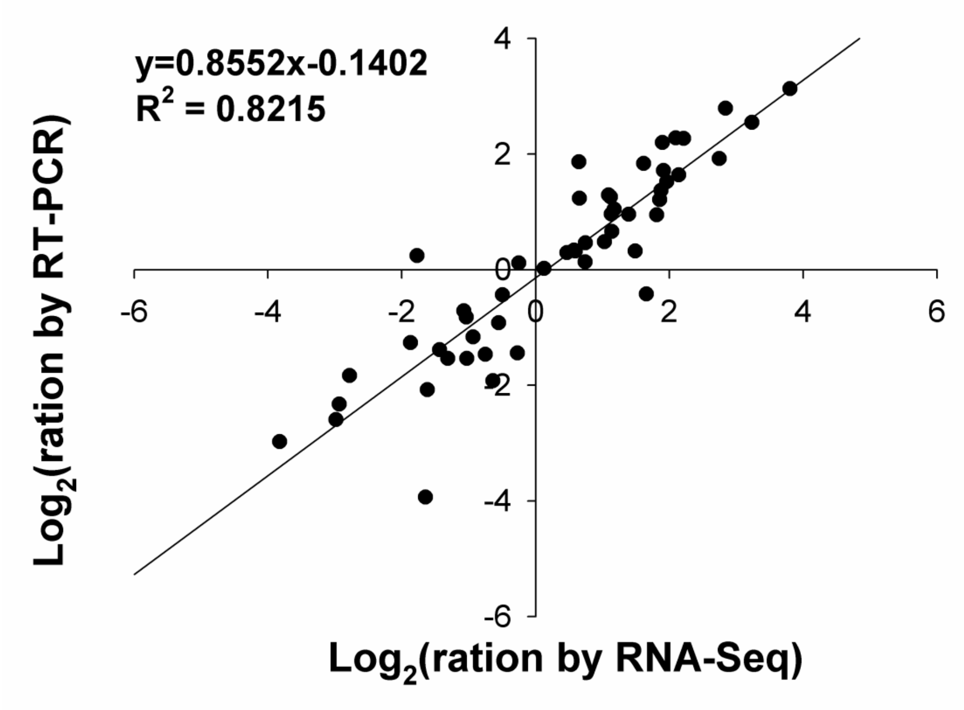


**Fig. S2** Comparison of the expression ratios of some selected genes using RNA-Seq and RT-PCR.

**Supplemental Table S1** Total number of sequencing reads and unique mapped reads obtained from each sample.

| Treatment | Total reads | Mapped reads | Unique mapped reads | Multiple mapped reads |
|---|---|---|---|---|
| **Leaf 0/0** | 11038533 | 8764490 | 8300678 | 463812 |
| **Leaf 100/100** | 10554680 | 8413039 | 7982602 | 430437 |
| **Leaf 0/200** | 11339866 | 9049574 | 8583943 | 465631 |
| **Root 0/0-0** | 10594575 | 8459664 | 7956384 | 503280 |
| **Root 100/100-100** | 9734566 | 7852209 | 7341002 | 511207 |
| **Root 0/200-0** | 10357301 | 8263282 | 7761111 | 502171 |
| **Root 0/200-200** | 9402266 | 7601605 | 7146244 | 455361 |

**Supplemental Table S2** The expression patterns of CK, ABA and ET biosynthesis and metabolic related genes in roots under uniform and non-uniform salinity treatments, which were significantly up- or down-regulated in the high-saline root sides.

| Hormones | Gene ID (Cotton_D_gene_) | $Log_2$ [(100/100-100) /(0/0-0)] | $Log_2$ [(0/200-0) /(0/0-0)] | $Log_2$ [(0/200-200) /(0/0-0)] | Gene annotation |
|---|---|---|---|---|---|
| Cytokinin | 10000877 | -1.4975 | 0.321928 | -5.58496 | Isopentenyltransferase 3, putative [*Theobroma cacao*] |
| | 10009222 | -1.65711 | 0.134301 | -3.03562 | Isopentenyltransferase 3, putative [*Theobroma cacao*] |
| | 10020909 | -2.45199 | -0.32193 | -4.37255 | Isopentenyltransferase 5, putative [*Theobroma cacao*] |
| | 10031541 | -1.27753 | 0.185867 | -3 | Isopentenyltransferase 3, putative [*Theobroma cacao*] |
| ABA | 10000877 | 1 | 0.415037 | 3.369234 | Nine-cis-epoxycarotenoid dioxygenase 3 [*Theobroma cacao*] |
| | 10009222 | 1.946229 | 0.286304 | 1.342888 | Nine-cis-epoxycarotenoid dioxygenase 3 [*Theobroma cacao*] |
| | 10020909 | 3.099536 | 1.280108 | 6.223312 | Nine-cis-epoxycarotenoid dioxygenase 3 [*Theobroma cacao*] |
| | 10031541 | 1.782409 | 0.400538 | 4.867896 | Nine-cis-epoxycarotenoid dioxygenase 5 [*Theobroma cacao*] |
| | 10032017 | -0.71811 | -0.28908 | -2 | ABA aldehyde oxidase isoform 1 [*Theobroma cacao*] |
| | 10032019 | -0.45873 | -0.15399 | -2.76648 | ABA aldehyde oxidase [*Theobroma cacao*] |
| | 10015829 | -0.49455 | -0.25445 | -1.86969 | ABA aldehyde oxidase isoform 1 [*Theobroma cacao*] |
| | 10003248 | 0.161895 | -1.62972 | 4.598605 | CYP707A2 [*Theobroma cacao*] |
| | 10016475 | 1.729511 | 0.898237 | 4.596633 | CYP707A4 isoform 1 [*Theobroma cacao*] |
| | 10024447 | 1.15614 | -0.2201 | 1.095445 | CYP707A1 [*Prunus persica*] |
| | 10029428 | 0.96131 | 0.284206 | 3.026753 | CYP707A4 isoform 2 [*Theobroma cacao*] |
| Ethylene | 10014605 | 2.745272 | 0.941314 | 6.0501 | ACC oxidase 3 [*Gossypium hirsutum*] |
| | 10023025 | 1.893719 | 0.574022 | 3.229989 | ACC oxidase 4 [*Gossypium hirsutum*] |
| | 10023026 | 1.683613 | 0.450377 | 3.771592 | ACC oxidase 4 [*Gossypium hirsutum*] |
| | 10033875 | -0.05071 | 0.273966 | -3.36409 | ACC oxidase 1 [*Gossypium hirsutum*] |
| | 10000894 | 1.697435 | 0.531507 | 3.936251 | ACC oxidase 3 [*Gossypium hirsutum*] |

**Supplemental Table S3** Summary of differentially expressed transcription factors (TFs) in roots of uniform and non-uniform salinity treatments.

| TF familay | Uniform salinity root (100/100-100) | | Non-saline root side (0/200-0) | | Hihg-saline root side (0/200-200) | |
|---|---|---|---|---|---|---|
| | Up-regulated | Dwon-regulated | Up-regulated | Dwon-regulated | Up-regulated | Dwon-regulated |
| NAC | 14 | 8 | 6 | 0 | 26 | 5 |
| ERF | 2 | 0 | 4 | 5 | 37 | 10 |
| WRKY | 5 | 2 | 1 | 0 | 16 | 2 |
| GRAS | 7 | 5 | 2 | 1 | 14 | 17 |
| MYB | 3 | 2 | 1 | 0 | 7 | 4 |
| Heat stress or shock | 2 | 2 | 0 | 0 | 4 | 8 |
| bZIP | 0 | 0 | 0 | 0 | 0 | 9 |
| Basic-leucine zipper | 0 | 3 | 0 | 0 | 3 | 4 |
| GATA | 0 | 8 | 0 | 0 | 4 | 10 |
| TCP | 0 | 0 | 0 | 0 | 0 | 5 |
| DREB | 0 | 1 | 0 | 0 | 3 | 0 |
| PLATZ | 3 | 0 | 0 | 0 | 3 | 1 |
| HD domain | 0 | 3 | 0 | 0 | 1 | 4 |
| bHLH | 0 | 1 | 0 | 0 | 3 | 1 |
| Nuclear Y subunit | 3 | 0 | 1 | 0 | 4 | 5 |
| Putative | 5 | 5 | 0 | 0 | 13 | 10 |
| Other | 3 | 1 | 1 | 0 | 6 | 10 |
| Total | 47 | 41 | 16 | 6 | 144 | 105 |

**Supplemental table S4** The expression patterns of TF genes in roots under uniform and non-uniform salinity treatments, which were significantly up- or down-regulated in the non-saline root sides.

| TF familay | Gene ID (Cotton_D_gene_) | $Log_2$ [(100/100-100)/(0/0-0)] | $Log_2$ [(0/200-0)/(0/0-0)] | $Log_2$ [(0/200-200)/(0/0-0)] | Gene annotation |
|---|---|---|---|---|---|
| NAC | 10008897 | 2.631198 | 1.016488 | 3.832405 | NAC domain protein, IPR003441 [*Theobroma cacao*] |
| | 10021542 | 0.459432 | 1.459432 | 2.285402 | Metallo-beta-lactamase family protein isoform 1 [*Theobroma cacao*] |
| | 10021859 | 1.087463 | 1.087463 | 3.044394 | NAC domain containing protein 90 [*Theobroma cacao*] |
| | 10037884 | 2.093361 | 1.612384 | 2.838589 | NAC domain protein, IPR003441 [*Theobroma cacao*] |
| | 10006239 | 2.314381 | 1.176878 | 4.294701 | NAC domain containing protein 47, putative isoform 2 [*Theobroma cacao*] |
| | 10015775 | 2.459432 | 1.321928 | 7.645658 | NAC domain protein, IPR003441 [*Theobroma cacao*] |
| ERF | 10024002 | -0.0039 | 1.022368 | -2.41504 | AP2/ERF transcription factor, putative [*Theobroma cacao*] |
| | 10001122 | 1.055874 | 1.361833 | 4.563965 | Ethylene-responsive transcription factor 1B [*Theobroma cacao*] |
| | 10007047 | 0.714736 | 1.234131 | 5.596353 | Ethylene-responsive transcription factor 1B [*Arabidopsis thaliana*] |
| | 10016954 | -0.30103 | 1 | 9.299208 | AP2/ERF transcription factor, putative [*Theobroma cacao*] |
| | 10031404 | -0.87918 | -1.20436 | -2.72792 | AP2/ERF transcription factor, putative [*Theobroma cacao*] |
| | 10015397 | -0.69897 | -1.09954 | 1.82103 | AP2/ERF transcription factor, putative [*Theobroma cacao*] |
| | 10010231 | -0.54407 | -1.22239 | 4.459432 | AP2/ERF transcription factor [*Theobroma cacao*] |
| | 10008036 | 0.295254 | -1.84206 | 3.757009 | Ethylene-responsive transcription factor 1B [*Arabidopsis thaliana*] |
| | 10038300 | -0.37447 | -2.54713 | 8.115409 | Ethylene-responsive transcription factor ERF109 [*Arabidopsis thaliana*] |
| WRKY | 10015331 | 1.986549 | 1.118502 | 2.683616 | WRKY transcription factor, putative [*Theobroma cacao*] |
| GRAS | 10016586 | 1.222564 | 1.056254 | 3.303761 | GRAS family transcription factor, putative [*Theobroma cacao*] |
| | 10017386 | 0.765287 | 1.415093 | 4.923511 | GRAS family transcription factor [*Theobroma cacao*] |
| | 10030105 | -3.83668 | -1.04639 | -5.55614 | GRAS family transcription factor [*Theobroma cacao*] |
| MYB | 10031822 | 1.678466 | 1.10591 | 3.125609 | Myb-like transcription factor family protein [*Theobroma cacao*] |
| Nuclear Y subunit | 10022082 | 2.001817 | 1.386675 | 1.902358 | Nuclear transcription factor Y subunit B-3 [*Theobroma cacao*] |
| Other | 10014462 | 1.881088 | 1.11369 | -0.48477 | C2H2L domain class transcription factor [*Theobroma cacao*] |

**Supplemental table S5** The expression patterns of genes in roots and leaves under uniform and non-uniform salinity treatments, as identified by RT-PCR.

| Gene ID (Cotton_D_gene_) | $Log_2$[(100/100-100)/(0/0-0)] | | $Log_2$[(0/200-0)/(0/0-0)] | | $Log_2$[(0/200-200)/(0/0-0)] | |
|---|---|---|---|---|---|---|
| | RNA-Seq | RT-PCR | RNA-Seq | RT-PCR | RNA-Seq | RT-PCR |
| **10012007** | 1.391 | 0.957 | 1.139 | 0.662 | 3.799 | 3.132 |
| **10005482** | 2.136 | 1.642 | -0.554 | -0.9177 | 2.212 | 2.272 |
| **10017573** | 0.655 | 1.234 | 0.646 | 1.865 | -1.772 | 0.242 |
| **10005406** | 1.875 | 1.373 | 0.744 | 0.461 | 1.913 | 1.714 |
| **10010732** | -2.936 | -2.323 | -1.028 | -1.536 | -3.824 | -2.973 |
| **10023025** | 1.894 | 2.198 | 0.574 | 0.338 | 3.230 | 2.547 |
| **10037884** | 2.093 | 2.281 | 1.612 | 1.836 | 2.839 | 2.793 |
| **10015829** | -0.495 | -0.435 | -0.254 | 0.117 | -1.87 | -1.163 |
| **10022762** | -0.27 | -1.439 | 0.469 | 0.292 | -1.649 | -3.936 |
| **10009753** | -0.934 | -1.163 | 0.738 | 0.135 | -2.983 | -2.594 |
| **10018577** | -1.315 | -1.536 | 1.027 | 0.485 | -2.78 | -1.829 |

| Gene ID (Cotton_D_gene_) | $Log_2$[(100/100)/(0/0] | | $Log_2$[(0/200)/(0/0] | |
|---|---|---|---|---|
| | RNA-Seq | RT-PCR | RNA-Seq | RT-PCR |
| **10014653** | -1.62 | -2.075 | 1.488 | 0.324 |
| **10031440** | -1.072 | -0.712 | 1.174 | 1.047 |
| **10014103** | -0.752 | -1.466 | 1.653 | -0.42 |
| **10021012** | -0.639 | -1.921 | 1.853 | 1.212 |
| **10038890** | 1.81 | 0.951 | 1.116 | 1.259 |
| **10001806** | 1.087 | 1.293 | 0.125 | 0.021 |
| **10007250** | 2.746 | 1.922 | 1.959 | 1.522 |
| **10004147** | 1.129 | 0.963 | 0.593 | 0.323 |
| **10007606** | -1.437 | -1.383 | -1.037 | -0.821 |

**Supplemental table S6** Primers used for RT-PCR analysis of genes described in Fig. 2 and Fig. 3.

| Gene name/Gene ID | Forward primer (5'– 3') | Reverse primer (5'– 3') |
|---|---|---|
| *β-actin* /FJ560483.1 | GATTCCGTTGTCCAGAAGTCCT | TACGGTCTGCAATACCAGGGA |
| *LHCB8*/CO075044.1 | CCAAGAATGAGGCTGGTGAGAT | GTGGTTGCCCTAAGTAAGATGATC |
| *PsbA1*/DW482288.1 | AGAGACACGAAAGCGAAATCCT | GAGCAGCAATGAATGCGACA |
| *PsbA*2/DN827239.1 | TCGCCTTCATTGTTGCTCCT | CAGATGCCGTTTCCCAGATC |
| *SOS1*/KM986873 | AAGCAGGTGATCTGAGTTGTAA | TGGGACAAGAAATCACAGCC |
| *SOS2*/GU188961.1 | ATCTGCAAATAAGGTTGGGGAA | GAAAAGGGTCAGCAAGTCAATG |
| *PMA1*/ES815719.1 | TCGTCGAACGTCCTGGACTT | TGTCCCAAGCCTTTCCACTAA |
| *PMA2*/JG838293.1 | AAGAAGAAGGGCAACGTCTCC | TTCTTGATCTCGTCGAGGCTG |
| *NHX1*/AF515632.2 | ACCTGGCACAATGTTACCGAG | AACAAGACCCATCAGCACAGC |
| *NHX6*/KM986874 | GGAGGCGTTCTTTGATCGTT | GCAAGTCCACCAACAATCAAAC |

**Supplemental table 7** Primers used for RT-PCR analysis of genes in Supplemental Table S5.

| Gene ID | Forward primer (5'– 3') | Reverse primer (5'– 3') |
|---|---|---|
| Cotton_D_gene_10012007 | AAACAGTCTACTCTAAGCCAACGC | TCAATGGCGGTATCAAAGGTT |
| Cotton_D_gene_10005482 | ACCCAAACGTACCACATCACATA | CAAATCCGTAACATCGCTTGGT |
| Cotton_D_gene_10017573 | TCTGGGCTATCAGGAGTGGATT | TTTTTCTTGCCTTCTCCATCTTT |
| Cotton_D_gene_10005406 | GATTTCTTGGGTCTTATCGGGG | GTCTTGAGGCTTTGAATCAGTCC |
| Cotton_D_gene_10010732 | ACAATGGAGTGCCGTAGGGAG | TGCAGCAAAGCCTCCTAATAAAC |
| Cotton_D_gene_10023025 | CGTTCTACAATCCCGGCAGC | GCCTGGAATTTAAGCAAAGCG |
| Cotton_D_gene_10037884 | GCTTTGACTATTTCCCTAACACCA | TATGACGGCTGACTAACTTGCTG |
| Cotton_D_gene_10015829 | GGACACCATTCCCAAACAATTC | GACTAATGTTGAACCAGCCTCG |
| Cotton_D_gene_10022762 | CATGCCAGTTCAAGATAGTCAAGAA | TTGGTTTTCTTCTCGGCGTC |
| Cotton_D_gene_10009753 | GACAGGCTCATCGCTCCAAT | TTATTCGCAAGACCATTCGTAGTT |
| Cotton_D_gene_10018577 | AATCAAGCCTCTTGTAGCAGCAT | AATGCTATTCCCACTGGAGTAGTC |
| Cotton_D_gene_10014653 | GCAACGGATGGAGCGAACA | ATTTGCCTCCTTCGCTACCA |
| Cotton_D_gene_10031440 | GTTGATAGGTTTGGGTCAGATCG | TTGCTGGTGATGTCTTGGAAAA |
| Cotton_D_gene_10014103 | AAGGTTATAGATTCGGTCAAGAGGA | ATAGCTAAAGCGATGGACCAGATA |
| Cotton_D_gene_10021012 | TCTTTAGACAGGGTTTCAGGGTCT | TGTAAGGGTCACCGCTCAGAT |
| Cotton_D_gene_10038890 | GTCCCACGAGTGTTCAATCTGTC | GTGGGTGGTATTGGTTGAGGTT |
| Cotton_D_gene_10001806 | GCCCCACATAAGAAGAACCAAA | CCTTGCTTTACCAACTCCCTTCT |
| Cotton_D_gene_10007250 | ACCCTCACTCTCCCCGTCAT | TGGCACGTAGGAACAAGGAAG |
| Cotton_D_gene_10004147 | AAGGATGATGTTTGAGAGGGAAAT | CAATGGAATCTGCTTGAACCCT |
| Cotton_D_gene_10007606 | GAGAGCCAAGCAAATGAACAGA | TCCCACATTTCAGGCACTCAT |
